# Analysis of Astragalus Polysaccharide Intervention in Heat-Stressed Dairy Cows’ Serum Metabolomics

**DOI:** 10.3390/ani10040574

**Published:** 2020-03-29

**Authors:** Hanfang Zeng, Yumeng Xi, Yeqing Li, Zedong Wang, Lin Zhang, Zhaoyu Han

**Affiliations:** 1 Institute of Dairy Science, College of Animal Science and Technology, Nanjing Agricultural University, Nanjing 210095, China; zenghanfang9303@163.com (H.Z.); 2016105006@njau.edu.cn (Y.L.); wangzedong@caas.cn (Z.W.); zhanglin9107@163.com (L.Z.); 2 Animal Husbandry Institute, Jiangsu Academy of Agricultural Sciences, Nanjing 210014, China; xiyumengxielei@163.com

**Keywords:** astragalus polysaccharides, heat stress, serum metabolomics, dairy cows

## Abstract

**Simple Summary:**

Heat stress reduces cow growth, milk yield and reproductive performance, and results in a significant economic loss for the dairy industry. Astragalus polysaccharides (APS), one major active ingredient of astragalus, have a wide range of biological activities. The results of the study revealed that APS has a significant effect on the levels of cortisol (COR), triiodothyronine (T3) and thyroxine (T4) in dairy cows’ serum under heat stress. Moreover, twenty metabolites were identified as potential biomarkers for the diagnosis of APS in heat-stressed dairy cows, and APS regulates the energy metabolism of heat stressed dairy cows through glucose metabolism and amino acid metabolism.

**Abstract:**

This experiment was conducted to investigate the effects of astragalus polysaccharides (APS) on serum metabolism of dairy cows under heat stress. Thirty healthy Holstein dairy cows were randomly divided into three groups (10 cows in each group). In the experimental group, 30 mL/d (Treatment I) and 50 mL/d (Treatment II) of APS injection were injected into the neck muscle respectively. Each stage was injected with APS for 4 days (8:00 a.m. every day) and stopped for 3 days. Serum hormone and antioxidant indexes of dairy cows were investigated. Through repeated measurement analysis of variance, the results have shown that cortisol (COR) (F = 6.982, *p* = 0.026), triiodothyronine (T3) (F = 10.005, *p* = 0.012) and thyroxine (T4) (F = 22.530, *p* = 0.002) at different time points were significantly different. COR showed a downward trend, T3 and T4 showed an upward trend. At each time point, different concentrations of APS have significant effects on COR (F = 30.298, *p* = 0.000 < 0.05), T3 (F = 18.122, *p* = 0.001), and T4 (F = 44.067, *p* = 0.000 < 0.05). However, there were no significant differences in serum insulin (INS), glucagon (GC) and heat shock protein 70 (HSP70) between different time points (*p* > 0.05) and at each time point (*p* > 0.05). Additionally, the results have also shown that there were also no significant differences in serum Superoxide dismutase (SOD), malondialdehyde (MDA) and lactate dehydrogenase (LDH) between different time points (*p* > 0.05) and at each time point (*p* > 0.05). However, the injection of APS had a significant impact on glutathione peroxidase (GSH-Px) (F = 9.421, *p* = 0.014) at different times, and showed a trend of rising first and then falling. At each time point, APS of different concentrations had no significant effect on GSH-Px (*p* > 0.05). Furthermore, we used gas chromatography–mass spectrometry (GC-MS) non-targeted metabolomics to determine the potential markers of APS for heat-stressed dairy cows. Twenty metabolites were identified as potential biomarkers for the diagnosis of APS in heat-stressed dairy cows. These substances are involved in protein digestion and absorption, glutathione metabolism, prolactin signaling pathway, aminoacyl-tRNA biosynthesis, pentose and glucuronate interconversions, and so on. Our findings suggest that APS have an effect on the serum hormones of heat-stressed dairy cows, and regulate the metabolism of heat-stressed dairy cows through glucose metabolism and amino acid metabolism pathways.

## 1. Introduction

Warming of the global climate environment limits the development of China’s dairy industry, especially in the high temperature and high humidity environment found in southern China. These environmental conditions cause cows to be under heat stress for a long time during the summer, ultimately leading to decreases in feed intake, reduced milk quality and reproduction [1,2]. Moreover, the incidence of mastitis in cows during the summer has increased, posing a serious challenge to dairy companies [3].

Most Chinese herbal medicines are extracted from pure natural green plants and contain a variety of bioactive substances. Previous studies have indicated that Chinese herbal medicine has a positive impact on the growth performance, immune response and serum metabolic index of livestock and poultry [4,5,6]. The study also showed that adding a particular fermented Chinese herbal medicine to the diet of dairy cows under high temperature stress could not only significantly improve milk yield, milk fat and milk protein, but also improve the immune function of dairy cows [7]. Furthermore, Chinese herbal medicine has few toxic side effects on animals, and is therefore a potential substitute for antibiotics in feed additives [8].

Astragalus is a major medicinal herb commonly used in many herbal formulations in the practice of traditional Chinese medicine [9]. A previous study indicated that dietary supplementation with 0.5% fermented Astragalus has beneficial effects on the growth performance, serum biochemical parameters, and fecal microbiota of broiler chickens [10]. A previous study also indicated that herbal medicine Huangqi decoction protected mice against chronic cholestatic liver injury and biliary fibrosis [11]. The major components of astragalus that have been identified are polysaccharides, flavonoids, saponins, and amino acids, and various biological activities of the compounds have been reported [9]. In recent years, scholars have paid attention to the biological value of astragalus polysaccharides (APS) [12,13]. Studies have shown that APS have good antioxidant activity both in vitro and *in vivo*. This is mainly achieved by improving the activity of superoxide dismutase (SOD) and glutathione peroxidase (GSH-Px) while reducing the production of malondialdehyde (MDA) [14,15]. Research shows that APS at higher doses (25 mg·mL^−1^) have stronger anti-tumor effects, decreasing more than 40.5% (24 h) and 67.3% (48 h) of liver cancer HepG2 cell viability. Thus, in vitro assays suggest that APS possess strong anti-tumor properties, which are beneficial for the treatment of liver cancer [16]. Moreover, evidence from previous research shows that APS inhibit cell growth and pro-inflammatory responses in IL-1beta-stimulated fibroblast-like synoviocytes [17]. APS also have a beneficial role in diabetes [18]. However, little is known about the potential mechanisms in dairy cows under heat stress.

Metabolic profiling, known as metabolomics, is increasingly used in clinical pharmacology and is an ideal tool for the acquisition of the several thousand metabolite alterations that are applied in the relationship between endogenous metabolite metabolism and body metabolism [19]. Metabonomics has been applied in cow science and has predicted the risk of diseases [20] and has been used for biomarker and pathway discovery in some metabolic diseases in cows [21]. A study has shown that 41 biomarkers in plasma metabolites of heat-stressed dairy cows have been identified. All these potential biomarkers are related to the metabolism of amino acids, lipids, carbohydrates, or intestinal microorganisms, which helps to clarify the physiological mechanism of heat stress-induced metabolic disorders and evaluate these biomarkers in practical applications [22]. Additionally, as potential biomarkers, glucose, lactate, pyruvate, lactose, β-hydroxybutyrate, citric acid, α-ketoglutarate, urea, creatine, and orotic acid, had high sensitivity and specificity for heat stress diagnoses. These substances are involved in glycolysis, and in lactose, ketone, tricarboxylic acid, amino acid and nucleotide metabolism, indicating that heat stress mainly affects lactose, energy and nucleotide metabolism of lactating dairy cows [23]. Therefore, metabonomics can provide a better understanding of the key metabolites, metabolic pathways, and major regulatory processes in heat-stressed dairy cows. However, how APS influences heat-stressed dairy cows remains undetermined. Therefore, identifying APS in heat-stressed cow serum metabolites and understanding their key pathways can deepen our understanding of APS in dairy cows.

Consequently, in order to analyze the effects of APS on heat-stressed dairy cows, we measured the serum hormones and antioxidant indexes in dairy cows. We also used non-targeted serum metabolomics to investigate the effects of APS on serum metabolism, and analyzed potential biomarkers of energy metabolism in heat-stressed dairy cows. These results might facilitate our understanding of the potential roles of APS in the intermediary metabolism of dairy cows under heat stress.

## 2. Material and Methods

### 2.1. Animals Ethics Statement

The procedures used in the present study were in compliance with the laws and regulations of China (based on the Animal Welfare Committee of Nanjing Agricultural University, China), and the internationally accepted principles and guidelines for the care and use of agricultural, laboratory, or experimental animals.

### 2.2. Animals and Experiment Design

Thirty healthy Holstein dairy cows were randomly divided into three groups (10 cows in each group). Information about parity, lactation days and milk yield of the cows is shown in Table 1. According to the amount of APS used in previous studies [24,25], in which APS is based on the content of glucose (C_6_H_12_O_6_) as the dose standard, 10 mL APS injections containing 0.1 g APS (Hebei Yuanzheng Pharmaceutical CO., LtD, Shijiazhuang, China), 30 mL/d (Treatment I) and 50 mL/d (Treatment II) of APS injection were injected into cows in this experiment, respectively. In order to facilitate the absorption of APS and reduce the stress response of dairy cows [26], we used neck muscular injection. Each stage was injected with APS for 4 days (8:00 a.m. every day) and stopped for 3 days. The experiment lasted for 21 days. In the whole experiment, we operated slowly, disinfected and stanched bleeding in time, and the cows did not show discomfort. The cows in the trial were fed three times daily, at ~0700, 1330 and 1900 h. The ingredients and composition of the experimental diets are presented in Table 2. Additionally, cows were allowed free access to water at all the time. Moreover, the cows were milked three times daily, at ~0800, 1430 and 2000 h. In addition, Treatment I was selected as a metabolomics study subject. Blood samples were collected from tail vein before (day 0, Q), during (day 4, Z) and after (day 21, H) to separate serum. Gas chromatography–mass spectrometry (GC-MS) was used to detect the changes of serum metabolites in dairy cows exposed to stress by non-targeted metabolomics (Shanghai Luming Biotechnology Co., Ltd., Shanghai, China).

### 2.3. Measuring and Sampling

Temperature (T, °C) and relative humidity (RH, %) in the cowshed were measured at 8:00, 14:00 and 20:00 every day during the experiment. Electronic thermometers at the six fixed points in the cowshed were read, respectively. The temperature humidity index (THI) criteria were derived from the NRC (1971), and THI was calculated according to THI= (1.8 × T + 32) − (0.55 − 0.55 × RH) × (1.8 × T−26), where T is the temperature (°C) and RH is the relative humidity (%) [27]. Rectal temperature and respiratory rate were measured by animal thermometer and stopwatch respectively on the 0, 4, 7, 11, 14, 18 and 21 days at 8:00 every day of the experiment. 

Blood samples (10 mL) were taken from the jugular vein before morning feedings at day 0, 4, 7, 11, 14, 18 and 21 of the trial period and collected into tubes until coagulation. The collected samples were centrifuged at 3000 r/min for 10 min and 4 °C, and stored at −20 °C subsequent serum hormone and antioxidant index measurements. Insulin (INS), glucagon (GC), cortisol (COR), triiodothyronine (T3), thyroxine (T4) and heat shock protein 70 (HSP70) were detected by ELISA assay kits (Shanghai Enzyme-linked Biotechnology Co., Ltd., Shanghai, China). Superoxide dismutase (SOD), malondialdehyde (MDA), glutathione peroxidase (GSH-Px) and lactate dehydrogenase (LDH) were quantified by enzymatic colorimetry using an AV400 Clinical Analyzer (Olympus Diagnostics, Tokyo, Japan), and reagents were supplied by Jiancheng Bioengineering Institute (Nanjing, China; catalog numbers A001-1, A003-1, A005 and A020-2). All assays were performed according to manufacturer’s instructions, and all serum samples were tested in triplicate.

### 2.4. Metabolite Extraction and Derivatization

All chemicals and solvents were analytical or HPLC grade. Methanol, acetonitrile, pyridine, n-hexane, methoxylamine hydrochloride, N, and O-Bis (trimethylsilyl) trifluoroacetamide (BSTFA) with 1% trimethylchlorosilane (TMCS) were purchased from CNW Technologies GmbH (Düsseldorf, Germany). L-2-chlorophenylalanine was from Shanghai Hengchuang Bio-technology Co., Ltd. (Shanghai, China). 

Serum samples stored at −80 °C were thawed at room temperature. A quantity of 80 μL of sample was added to a 1.5 mL Eppendorf tube with 20 μL of 2-chloro-l-phenylalanine (0.3 mg/mL) dissolved in methanol as internal standard, and the tube was vortexed for 10 s. Subsequently, 240 μL of ice-cold mixture of methanol and acetonitrile (2/1, v/v) was added, and the mixtures were vortexed for 1 min, ultrasonicated at ambient temperature (25 °C to 28 °C) for 5 min, and stored at −20 °C for 10 min. The extract was centrifuged at 12000 rpm, 4 °C for 10 min. The samples were centrifuged at 12000 rpm for 10 min at 4 °C. A QC sample was prepared by mixing aliquots of all the samples to be a pooled sample. The inter- and intra-assay coefficients of variation were below 30 %. An aliquot of the 150 μL supernatant was transferred to a glass sampling vial for vacuum drying at room temperature. A quantity of 80 μL of 15 mg/mL methoxylamine hydrochloride in pyridine was subsequently added. The resultant mixture was vortexed vigorously for 2 min and incubated at 37 °C for 90 min. A quantity of 80 μL of BSTFA (with 1% TMCS) and 20 μL n-hexane was added into the mixture, which was vortexed vigorously for 2 min and then derivatized at 70 °C for 60 min. The samples were placed at ambient temperature for 30 min before GC-MS analysis. Chromatography analysis and identification were performed as previously described by Lu et al. (2018) [28].

### 2.5. Statistic Analysis

The experimental data were analyzed by repeated measures analysis of variance by SPSS 20.0, and data were considered statistically significant when *p* < 0.05. The results of all data were expressed by Mean (+SEM). 

ChemStation (version E.02.02.1431, Agilent, USA) software was used to convert the raw data (D format) to Common Data Format (CDF), and then the CDF data were imported into the ChromaTOF software (version 4.34, LECO, St Joseph, MI, USA) for data processing. Metabolites were annotated through the Fiehn or NIST database. After alignment with Statistic Compare component, the ‘raw data array’ (.cvs) was obtained from raw data with three dimension data sets including sample information, peak names (or retention time and m/z) and peak intensities. There were 728 peaks detected from all samples. In the ‘data array’, all internal standards and any known pseudo positive peaks (caused by background noise, column bleed or BSTFA derivatization procedure) were removed. The data was normalized to the total peak area of each sample, and multiplied by 10000, and the peaks from the same metabolite were combined. The total detectable metabolites were 227. Data were transformed by log2 in Excel 2007 (Microsoft, USA) (use 0.000001 to replace 0 before transforming), and the resulting data matrix were then imported into SIMCA software package (14.0, Umetrics, Umeå, Sweden). Principal component analysis (PCA) and orthogonal partial least-squares discriminant analysis (OPLS-DA) were performed to visualize the metabolic difference among experimental groups, after mean centering and unit variance scaling. The Hotelling’s T2 region, shown as an ellipse in score plots of the models, defines the 95 % confidence interval of the modeled variation. Variable importance in the projection (VIP) ranks the overall contribution of each variable to the OPLS-DA model, and those variables with VIP > 1 are considered relevant for group discrimination. The differential metabolites were selected on the basis of the combination of a statistically significant threshold of VIP values obtained from the OPLS- DA model and p values from a two-tailed Student’s t-test on the normalized peak areas from different groups (Z *vs* Q, H *vs* Z, H *vs* Q), where metabolites with VIP values larger than 1.0 and p values less than 0.05 were considered as differential metabolites. The fold change (FC) of different metabolites in the two groups was calculated. The multiple of change is the ratio of the average content of the differential metabolite in the two groups.

## 3. Results

### 3.1. Environmental Temperature, Relative Humidity and Temperature and Humidity Index of Cowshed

**Figure 1:**
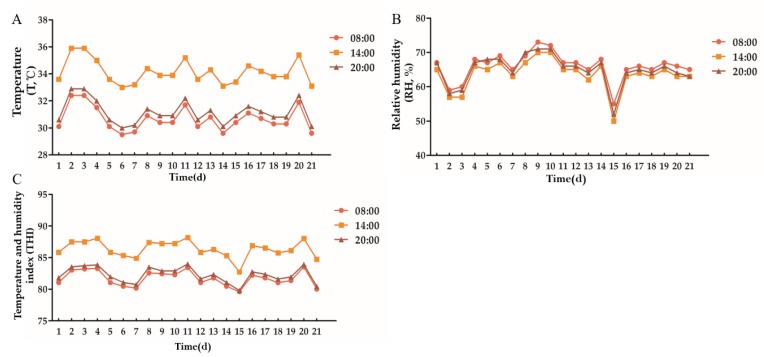
Environmental temperature, relative humidity and temperature and humidity index of cowshed. (**A**): Temperature, (**B**): Relative humidity, (**C**): Temperature and humidity index.

According to Figure 1, the environmental temperature of the cowshed varied between 33.0 °C and 35.9 °C, the relative humidity varied between 48% and 70%, and the THI varied between 82.36 and 88.17 during the experiment. Ingraham et al. (1974) believed that THI < 72 had no effect on dairy cows. It would cause slight heat stress on dairy cows between 72 and 78, moderate heat stress on dairy cows between 78 and 89, and severe heat stress on dairy cows above 90 [29]. Therefore, in the current experiment, dairy cows are in a state of moderate heat stress.

### 3.2. Rectal Temperature and Respiratory Rate of Dairy Cows

Table 3 shows the rectal temperature and respiratory rate of the cows during the experiment. In general, the rectal temperature of cows was 38.5 °C in normal conditions, and the respiratory rate was 18~28 times·min^−1^ in quiet conditions. In our experiment, the rectal temperature of dairy cows was higher than 38.5 °C, and the respiratory rate was higher than 40 times·min^−1^. Therefore, in the absence of other diseases, cows are in a state of heat stress.

### 3.3. Serum Hormone, HSP70 and Antioxidant Index

As shown in Table 4, after injection of APS, there were no significant differences in serum INS (F = 0.199, *p* = 0.950), GC (F = 0.245, *p* = 0.925), and HSP70 (F = 3.440, *p* = 0.101) between different time points. COR (F = 6.982, *p* = 0.026), T3 (F = 10.005, *p* = 0.012) and T4 (F = 22.530, *p* = 0.002) at different time points showed significant differences. COR showed a downward trend, T3 and T4 showed an upward trend. At each time point, APS of different concentrations had no significant effect on INS (F = 0.416, *p* = 0.670), GC (F = 0.443, *p* = 0.655) and HSP70 (F = 0.182, *p* = 0.836). However, different concentrations of APS have significant effects on COR (F = 30.298, *p* = 0.000 < 0.05), T3 (F = 18.122, *p* = 0.001), and T4 (F = 44.067, *p* = 0.000 < 0.05). Among them, APS with different concentrations had significant effects on COR on the 4th, 18th and 21st day, and had significant effect on T3 on the 11th and 14th day (*p* < 0.05). In addition, different concentrations of APS had an effect on T4 during the experimental period (*p* < 0.05). Moreover, there was no interaction between INS, GC, COR, T4 and HSP70 at different times and concentrations (*p* > 0.05), but T3 had an interaction effect on APS at different times and concentration.

As shown in Table 5, after injection of APS, there were no significant differences in serum SOD (F = 0.692, *p* = 0.652), MDA (F = 1.239, *p* = 0.410) and LDH (F = 3.440, *p* = 0.101) between different time points. However, the injection of APS had a significant impact on GSH-Px (F = 9.421, *p* = 0.014) at different times, and showed a trend of rising first and then falling. At each time point, APS of different concentrations had no significant effect on SOD (F = 0.468, *p* = 0.641), MDA (F = 0.572, *p* = 0.584), GSH-Px (F = 0.922, *p* = 0.432) and LDH (F = 0.182, *p* = 0.836). Moreover, there was no interaction between SOD, MDA and LDH at different times and concentrations (*p* > 0.05), but GSH-Px had an interaction effect on APS at different times and concentrations.

### 3.4. Metabolomics Analysis of Dairy Cow Serum

**Figure 2:**
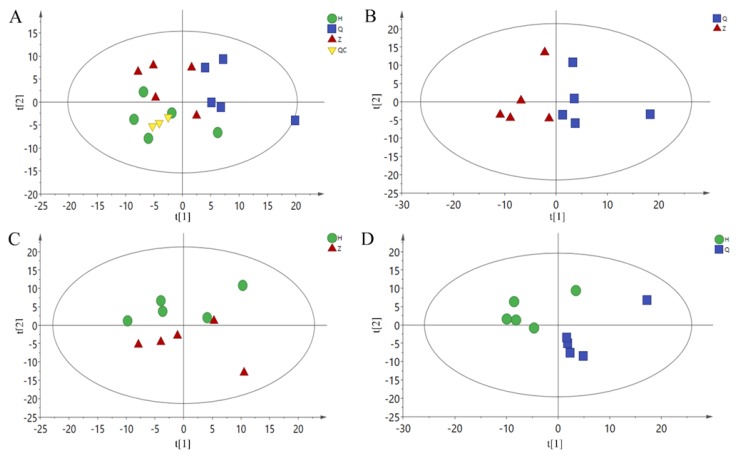
PCA Scores plot. (**A**): All-PCA Scores plot, (**B**): Z vs Q PCA Scores plot, (**C**): H vs Z PCA Scores plot, (**D**): H vs Q PCA Scores plot. Q: Pre-test, Z: Mid-test, H: Post-test, QC: quality control.

A total of 227 metabolites were identified by GC-MS. Metabonomic data were imported into the SIMCA software package (14.0, Umetrics, Umeå, Sweden). As shown in Figure 2A, the PCA model shows that all samples are basically within the 95% confidence interval. There is a trend of separation among the three groups of samples. Because of individual differences between samples, the results of principal component analysis are not completely separated, indicating that the constructed model is stable and reliable. In order to further obtain the metabolic differences between the two groups, the PCA scores of the two groups were compared with those of the mid-test and pre-test (Z vs Q), post-test and mid-test (H vs Z), and post-test and pre-test (H vs Q), as shown in Figure 2B–D. Comparing the two groups of samples, there is a trend of separation, and there is no overlap between the three groups. Therefore, the current PCA model can be used to explain the metabolic differences between the two groups more reliably.

**Figure 3:**
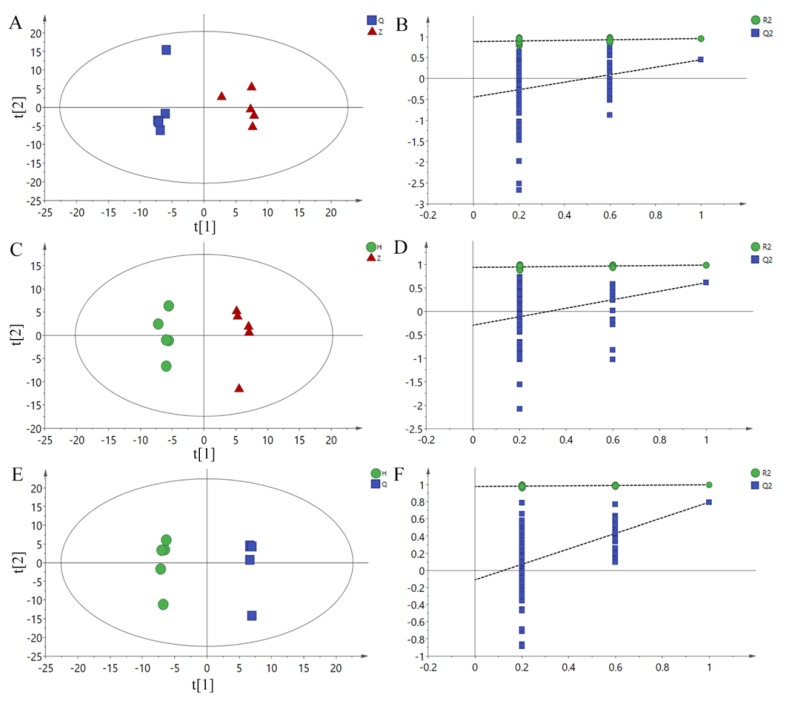
OPLS-DA scores plots and permutation test plots. (**A**): Z vs Q PLS-DA Scores plot, (**B**): H vs Z PLS-DA scores plot, (**C**): H vs Q PLS-DA scores plot. (**D**): Z vs Q permutation test plots, (**E**): H *vs* Z permutation test plots, (**F**): H *vs* Q permutation test plots. Q: Pre-test, Z: Mid-test, H: Post-test.

In order to obtain more reliable differential metabolite analysis and filter signals unrelated to model classification, an OPLS-DA model was obtained by using OPLS-DA between Z vs Q, H vs Z and H vs Q groups, and RPT with 200 responses was used. The parameters of each model are shown in Table 6. In the RPT test, Q^2^ should be less than zero under normal conditions. Both R^2^ and Q^2^ suggested that the model was effective. As shown in Figure 3A,C,E, the two groups of samples were separated on the OPLS-DA scores plots, which shows that the current model can explain the differences between groups very well. Therefore, the above pattern discrimination can be used for the screening of potential biomarkers.

### 3.5. Screening of Differential Metabolites

**Figure 4:**
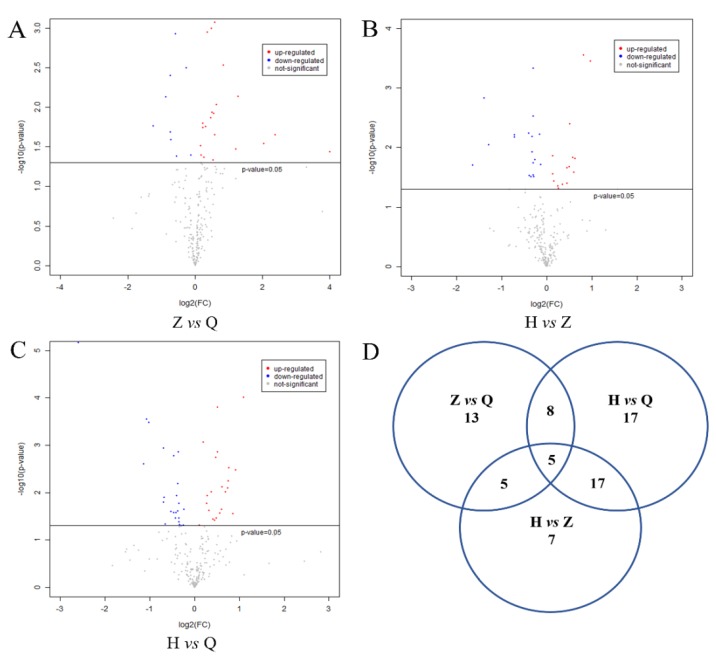
Expression of differential metabolites in serum of dairy cows and Venn diagram. Note: (**A–C**) were volcanic plots of Z vs Q, H vs Z and H vs Q, respectively. *−*log10(*p*-value) is from the original *p*-value calculated from the differential metabolites. *−*log10 (0.05) = 1.30 *−* log10 (*p*-value) > 1.30 means significant difference. The red dot indicates log2FC > 0, and the metabolite is upregulated. The blue dot indicates log2FC < 0, and the metabolite is downregulated. The gray dot means no significant difference (*−*log10 (*p*-value) < 1.3). (**D**) is the Ven diagram of the difference variables between groups.

The metabolites were assigned according to the Fiehn database, and the FC values of the differential metabolites in the two groups were calculated. After log2 conversion, log2FC > 0 indicates upregulation and log2FC < 0 indicates downregulation. As shown in Figure 4A–C, −log10(*p*-value) is from the original *p*-value calculated from the differential metabolites. −log10 (0.05) = 1.30. −log10 (*p*-value) > 1.30 means significant difference. The red dot indicates log2FC > 0, and the metabolite is upregulated. The blue dot indicates log2FC < 0, and the metabolite is downregulated. The gray dot means no significant difference (−log10 (*p*-value) < 1.3). As shown in Figure 4D, a total of 31 differential metabolites were obtained between the mid-test and pre-test, 47 differential metabolites were observed between the post-test and mid-test, and 34 differential metabolites were detected between the post-test and pre-test.

### 3.6. Identification and Comparison of Differential Metabolites

**Figure 5:**
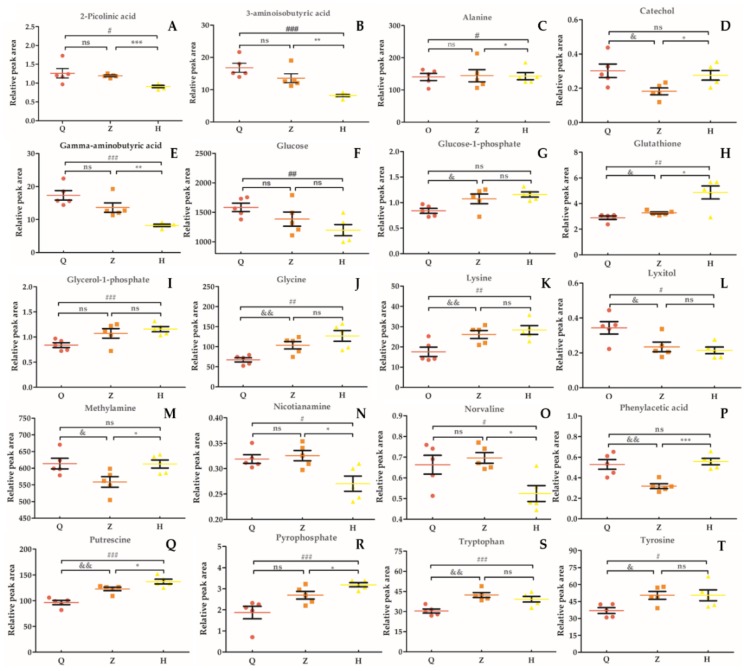
Relative peak area of serum marker metabolites in dairy cows. (**A**): 2-Picolinic acid. (**B**): 3-Aminoisobutyric acid. (**C**): Alanine. (**D**): Catechol. (**E**): Gamma-aminobutyric acid. (**F**): Glucose. G: Glucose-1-phosphate. (**H**): Glutathione. (**I**): Glycerol-1-phosphate. (**J**): Glycine. (**K**): Lysine. (**L**): Lyxitol. (**M**): Methylamine. (**N**): Nicotianamine. (**O**): Norvaline. (**P**): Phenylacetic acid. (**Q**): Putrescine. (**R**): Pyrophosphate. (**S**): Tryptophan. (**T**): Tyrosine. “&” means Z vs Q significant difference (*p* < 0.05), “*” means H vs Z significant difference (*p* < 0.05), “#” means H vs Q significant difference (*p* < 0.05); “ns” means the difference is not significant (*p* > 0.05).

**Figure 6:**
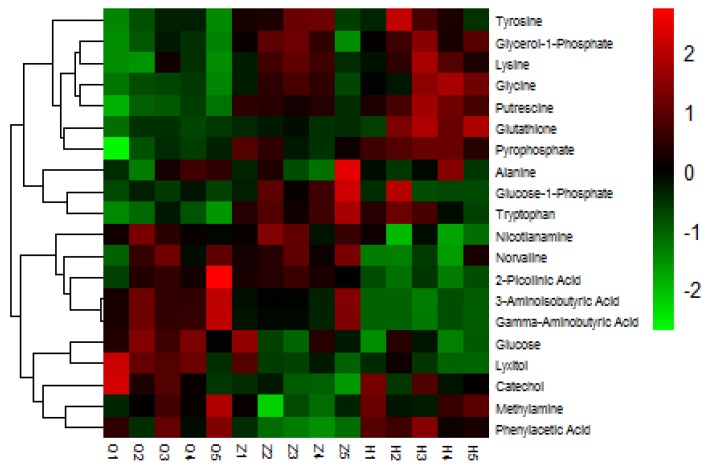
Heat map of serum differential metabolites in dairy cows. Changes in color represent changes in relative peak area, where red represents increased serum metabolite content and blue represents decreased serum metabolite content.

In this study, we selected 20 different metabolites with biomarkers in cow serum; their retention time, quant mass and classification are shown in Table 7, and their peak areas are shown in Figure 5. Among these metabolites, glucose-1-phosphate, glutamine, glycerol-1-phosphate, glycine, lysine, pyrophosphoric acid, putrescine, tryptophan, and tyrosine were upregulated before and after the test. 2-picolinic acid, 3-aminoisobutyric acid, alanine, γ-aminobutyric acid, glucose, sugar alcohol, nicotinamide, norvaline, and phenylacetic acid were downregulated before and after the test. Catechol and methylamine showed a downward trend after being upregulated after injection of APS. As shown in Figure 6, in order to more intuitively and clearly understand the changes of 20 differential metabolites in dairy cow serum, cluster analysis was performed on the peak area.

### 3.7. Differential Metabolite Metabolic Pathway

**Figure 7:**
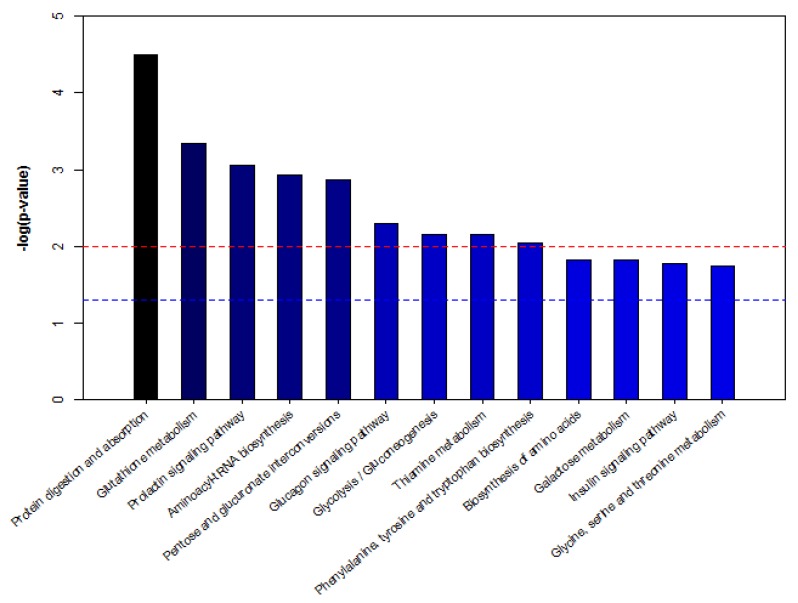
Pathway analysis of metabolites from dairy cows’ serum. Note: −log (*p*) is from the original *p*-value calculated from the enrichment analysis. The red line indicates *p* = 0.01, and the blue line indicates *p* = 0.05. When the column top is higher than the blue line or the red line, the signal path is significant.

To further study the biomarkers, we obtained 20 biomarkers’ KEGG IDs ((Kyoto encyclopedia of genes and genomes identifications) and enriched the serum potential biomarkers of dairy cows by MBROLE (Metabolites biological role) pathway analysis. As shown in Figure 7, according to *p* < 0.05 in metabolic pathway, the enrichment map of metabolic pathway is drawn with the name of metabolic pathway as abscissa and −log (*p*-value) as ordinate. The metabolic pathways with −log (*p*) > 1.5 are considered the most relevant pathways involved in the conditions under experiment. Among these pathways, biological modules were involved in sugar metabolism, protein metabolism, nucleotide metabolism and vitamin metabolism. These include protein digestion and absorption, glutamine metabolism, the prolactin signaling pathway, aminoacyl-tRNA biosynthesis, pentose and glucuronate interconversions, the glucagon signaling pathway, glycolysis/gluconeogenesis, thiamine metabolism, phenylalanine, tyrosine and tryptophan biosynthesis, galactose metabolism, the insulin signaling pathway, and glycine, serine and threonine metabolism, etc. Hence, five metabolic pathways were selected as potential metabolic pathways for APS, affecting serum metabolism of heat-stressed cows (Table 8).

## 4. Discussion

Recently, APS has drawn extensive attention because of its immunity, antiviral, hypoglycemic, and antioxidant properties [30,31,32]. However, the mechanism of application in heat-stressed dairy cows is not known. Generally, heat stress not only reduces growth, reproductive efficiency, milk yield, and milk quality [33], but also affects the hormone metabolism of dairy cows [34]. COR is a stress hormone secreted by the adrenal cortex. In this study, the level of COR in the experimental group increased after APS injection. Christison et al. (1972) proved that the plasma COR of dairy cows increased significantly in a state of acute heat stress. However, the short- and long-term environmental heat effects on COR are clear, with initial increases due to acute stressors and a decline of amounts in plasma levels after prolonged exposure to stressors [35]. Furthermore, the secretion of COR is closely related to milk production and physiological metabolism compensation [36], which may have led to our results. T3 and T4 are secreted by the thyroid gland and its peripheral tissues. The cell signals mediated by T3 and T4 play a key role in regulating body temperature, energy intake, and metabolism [37]. When the body is exposed to a thermal environment, its basic metabolic rate and heat production will be reduced, and the synthesis and secretion of T3 and T4 in the thyroid of dairy cows will be reduced [38]. Our results showed that T3 and T4 levels increased significantly after APS injection. This might be because APS can recover the metabolism of dairy cows by increasing the concentration of T3 and T4, and alleviating the heat stress of dairy cows. Previous studies revealed that a Chinese herbal medicine additive can increase the concentration of T3 and T4 in the serum of heat-stressed cows, reduce the concentration of COR and adrenocorticotropic hormone, and alleviate heat stress by regulating the serum hormones and enzymes of cows [39]. This result was in accordance with the present results on T3 and T4. Moreover, in this study, our results showed that APS had no significant effect on INS and GC in heat-stressed dairy cows. Generally, heat stress results in higher insulin secretion in lactating cows [40]. Research has shown that dietary supplementation with traditional Chinese medicine can reduce the serum glucose level and increase the apparent nutrient digestibility of heat-stressed beef cattle [41]. However, adding Chinese herbal medicine to a basic diet improves the serum glucose level of chickens under heat stress conditions [42]. These results may be related to the secretion of insulin and glucagon in different states. Our results showed that APS had no effect on the secretion of insulin and glucagon under heat stress. This may be related to the intake and milk production of cows.

Additionally, the production rate of oxygen free radicals is higher than the clearance rate due to the high metabolic rate of cows under heat stress conditions, especially in lactating cows. Consequently, the antioxidative function of dairy cows was decreased [43]. In present study, the results showed that APS had no significant effect on the serum antioxidant index of dairy cows. However, APS also did not cause an abnormal antioxidant index. This may be due to the long-term heat stress state of dairy cows, and its antioxidant level may have reached a balance, but the injection of APS in a short time is not sufficient to change its serum antioxidant level. HSP70 also has no significant effect and may be caused by the above reasons. Although astragalus as an adjunctive treatment to conventional therapies was found to offer some promising effects in serum components, suboptimal methodological quality and poor reporting meant that definitive conclusions could not be made based on available evidence [44].

In order to analyze the effects of APS on the metabolism of heat-stressed dairy cows, the metabolomics method based on GC-MS was used to determine the changes in serum. Our results showed that 294 metabolites were detected in the serum of dairy cows. After PCA analysis and OPLS-DA analysis, 20 biomarkers were identified, and the comparison among groups was statistically significant. These biomarkers are mainly involved in the metabolism of serum sugar, amino acids, and vitamins in dairy cows. They are potential markers the effects of APS on dairy cows.

In our study, glucose-1-phosphate was upregulated, and 3-aminoisobutyric acid, glucose, and glycol were downregulated in the serum of dairy cows after APS injection, and catechol was significantly increased after APS injection. Previous studies have shown that APS is particularly prominent in the treatment of diabetes mellitus, and it is a candidate for insulin resistance therapy [45,46]. Glucose-1-phosphate is the substrate of glycosyl derivatives [47], and it is also an important glycogen synthesized by liver and muscle in adrenal dysfunction [48]. β-aminoisobutyric acid, also known as DL-3-aminoisobutyric acid, is produced in skeletal muscle during exercise. The study has shown that β-aminoisobutyric acid plays a role in diabetes [49]. It has also been confirmed that β-aminoisobutyric acid can attenuate lipopolysaccharide-induced inflammation and insulin resistance in adipocytes through AMP-activated protein kinase-mediated pathways [50]. Catechol belongs to the catecholamines and is a precondition for the synthesis of epinephrine and norepinephrine [51]. Heat stress can significantly increase the levels of serum adrenaline and noradrenaline levels [52]. Moreover, in a study of compensatory changes in glucose production and utilization after insulin administration, it was found that acute glucocorticoid is glucagon and catecholamine. When glucagon secretion is impaired, the adrenergic mechanism, especially adrenomedullary adrenaline, is essential for recovery from hypoglycemia [53]. Therefore, our results show that these substances may affect the level of serum glucose metabolism in dairy cows by affecting the glycolysis/gluconeogenesis process, insulin signaling pathway and glucagon signaling pathway. Thus, this alleviates the imbalance of energy metabolism caused by heat stress in dairy cows.

In addition, after APS injection, serum glutamine, glycine, lysine, tryptophan and tyrosine were upregulated. However, alanine and valine were downregulated in the current study. Alanine, glutamine, glycine, and proline are all raw sugar amino acids, lysine is a ketogenic amino acid, and tryptophan and tyrosine are raw sugar ketone amino acids. According to a previous study, after 24 h of discontinuation of insulin administration, the levels of alanine, glycine and threonine in the arteries of diabetic patients decreased, but the levels of valine, leucine and isoleucine increased [54]. Moreover, the serum levels of valine, alanine, tyrosine and tryptophan in type 2 diabetes mellitus patients were increased by using serum metabonomics [55]. Therefore, our results further prove that APS can regulate the amino acid metabolism pathway, intervene in the metabolic disorder of cows under stress, and reduce the physiological effect of stress in the summer.

γ-Aminobutyric acid (GABA) is an inhibitory neurotransmitter produced by the central nervous system, which affects energy metabolism in the brain [56]. The results showed that the addition of rumen-protective GABA (RP-GABA) to beef cattle’s diet could significantly increase the average daily intake of heat-stressed beef cattle, and improve the nutritional digestibility, growth performance and antioxidant capacity of heat-stressed beef cattle [57]. Nicotinamide is an amide form of nicotinic acid (vitamin B3), which participates in cell proliferation and differentiation [58] and energy metabolism [59] and can improve energy metabolism diseases [60]. Additionally, inducing the use of pyrophosphate (PPI) as an energy enzyme to replace the use of ATP is a potential strategy to deal with the energy crisis [61]. Our study showed that GABA and nicotinamide were downregulated and pyrophosphate was upregulated after APS injection. Therefore, APS plays an important role in the energy metabolism of heat-stressed dairy cows.

## 5. Conclusions

In summary, the current results showed that APS had a significant effect on the levels of COR, T3 and T4 in dairy cows’ serum under heat stress. After APS injection, glucose-1-phosphate, glutamine, glycerol-1-phosphate, glycine, lysine, pyrophosphoric acid, putrescine, tryptophan, and tyrosine were upregulated. 2-picolinic acid, 3-aminoisobutyric acid, alanine, γ-aminobutyric acid, glucose, sugar alcohol, nicotinamide, norvaline, and phenylacetic acid were downregulated. Catechol and methylamine showed a downward trend after being upregulated after injection of APS. Consequently, APS regulates the energy metabolism of heat stressed dairy cows through glucose metabolism and amino acid metabolism.

## Figures and Tables

**Table 1:** Characteristics of the Holstein dairy cows used in the study.

Parameter	CON	Treatment I	Treatment II	*p*-value
Number of cows	10	10	10	
Parity	1.88 ± 0.83	2.00 ± 0.75	1.75 ± 0.89	0.83
Lactation days(d)	211.25 ± 43.21	217.00 ± 44.28	195.25 ± 59.10	0.42
Mike yield (kg/d)	34.62 ± 4.78	34.62 ± 5.62	34.62 ± 4.07	1.00

Note: CON, control group; Treatment I, 30 mL/d APS injection; Treatment II, 50 mL/d APS injection.

**Table 2:** Composition and nutrient levels of basal diet of dairy cows (DM basis) %.

Item	Content(%)	Item	Content
Silage corn	17.47	Nutrient levels ^(2)^	
Alfalfa grass	20.66	DM (%)	57.10
Corn flour	21.01	ME (MJ/kg)	11.16
Cardamom	8.00	NE_L_ (MJ/kg)	7.20
Oatgrass	5.02	CP (%DM)	17.40
Wet brewer’s grains	4.53	NDF (%)	30.85
Beet pulp	5.13	ADF (%)	19.59
Cottonseed	4.68	EE (%)	5.56
Rapeseed meal	1.20	Ash (%)	7.73
Puffed soybean	3.95	Ca (%)	0.65
Tablet corn	2.40	P (%)	0.45
DDGS	2.43		
Premix ^(1)^	1.05		
Fatty acid calcium	0.43		
Calcium hydrogen phosphate	0.50		
Baking soda	0.74		
Salt	0.50		
MgO	0.30		
Total	100.00		

^(1)^ The premix provided the following per kg of diets: VA 5 130 IU, VD_3_ 1 283 IU, VE 26 mg, biotin 0.05 mg, β-carotene 0.10 mg, Mn 12 mg, P 12 mg, S 0.85 mg, Zn 64 mg, Se 0.4 mg, Co 0.19 mg. ^(^^2)^ Nutrient levels.

**Table 3:** Rectal temperature and respiratory rate of dairy cows.

Item	Time(d)	Groups		
		CON	Treatment Ⅰ	Treatment Ⅱ
Rectal temperature (°C)	0	38.74 ± 0.08	38.89 ± 0.06	38.74 ± 0.08
	4	39.11 ± 0.15	38.73 ± 0.08	38.80 ± 0.07
	7	39.35 ± 0.11	39.04 ± 0.06	39.12 ± 0.09
	11	39.25 ± 0.09	39.10 ± 0.13	38.76 ± 0.07
	14	39.43 ± 0.10	39.10 ± 0.04	39.08 ± 0.07
	18	39.28 ± 0.08	39.16 ± 0.06	38.88 ± 0.08
	21	39.44 ± 0.13	38.85 ± 0.06	38.70 ± 0.06
Respiratory rate (times·min^−1^)	0	47.75 ± 2.18	38.87 ± 1.78	32.38 ± 1.93
	4	57.50 ± 4.14	53.50 ± 3.54	39.25 ± 3.69
	7	49.88 ± 3.80	47.00 ± 6.15	45.50 ± 5.72
	11	53.50 ± 3.62	45.75 ± 3.03	38.00 ± 5.01
	14	62.00 ± 8.23	55.75 ± 3.92	37.25 ± 3.54
	18	61.25 ± 3.71	48.75 ± 7.77	49.25 ± 3.37
	21	61.25 ± 5.25	48.75 ± 2.75	47.50 ± 5.14

**Table 4:** Effects of astragalus polysaccharides on serum hormone and HSP70 of heat-stressed dairy cows.

Item	Groups	Time (d)							Holistic Analysis (Huynh-Feldt)	Concentration (F, P)	Time (F, P)	Interaction (F, P)
		0	4	7	11	14	18	21				
INS/(μIU/L)	CON	20.24 ± 2.57	17.20 ± 2.69	17.14 ± 3.11	17.93 ± 2.75	15.43 ± 2.90	16.26 ± 2.73	15.85 ± 3.28	1.000	0.416, 0.670	0.199, 0.950	1.102, 0.446
	Treatment Ⅰ	17.87 ± 4.90	14.31 ± 1.77	14.38 ± 3.06	14.16 ± 1.97	16.35 ± 3.61	15.04 ± 3.17	18.84 ± 4.03				
	Treatment Ⅱ	17.09 ± 3.31	16.84 ± 2.87	18.35 ± 1.53	18.49 ± 3.53	21.31 ± 3.19	16.29 ± 2.50	17.39 ± 1.40				
*p*-value		0.321	0.697	0.856	0.529	0.621	0.492	0.492				
GC/(pg/mL)	CON	96.06 ± 8.83	107.31 ± 14.52	107.31 ± 14.52	122.58 ± 13.18	97.74 ± 18.71	95.54 ± 12.73	99.10 ± 15.33	1.000	0.443, 0.655	0.245, 0.925	2.008, 0.210
	Treatment Ⅰ	114.05 ± 10.35	103.95 ± 16.06	103.95 ± 16.06	104.99 ± 17.10	120.38 ± 9.71	114.95 ± 16.12	88.88 ± 14.60				
	Treatment Ⅱ	103.95 ± 15.18	95.54 ± 11.97	95.54 ± 11.97	115.47 ± 9.61	108.22 ± 7.05	107.51 ± 15.21	114.82 ± 8.14				
*p*-value	0.576	0.838	0.668	0.487	0.226	0.407	0.407	0.576				
COR(ng/mL)	CON	60.77 ± 2.86	57.91 ± 2.71	57.55 ± 2.46	49.97 ± 2.21	48.35 ± 2.02	48.22 ± 3.35	47.31 ± 1.63	1.000	30.298, 0.000	6.982, 0.026	1.976, 0.216
	Treatment Ⅰ	66.61 ± 2.06	57.28 ± 2.69	56.84 ± 3.42	54.01 ± 2.98	49.87 ± 2.75	53.12 ± 1.86	51.92 ± 2.80				
	Treatment Ⅱ	66.04 ± 1.65	66.39 ± 1.16	60.85 ± 1.97	60.08 ± 2.25	58.90 ± 2.61	62.35 ± 2.44	62.11 ± 2.42				
*p*-value		0.184	0.037	0.135	0.094	0.068	0.004	0.004				
T3 (nmol/L)	CON	2.10 ± 0.06	2.03 ± 0.09	2.03 ± 0.09	2.03 ± 0.07	2.10 ± 0.02	2.33 ± 0.04	2.32 ± 0.08	1.000	18.122, 0.001	10.005, 0.012	7.338, 0.015
	Treatment Ⅰ	2.18 ± 0.10	2.27 ± 0.12	2.27 ± 0.11	2.23 ± 0.03	2.43 ± 0.12	2.48 ± 0.10	2.50 ± 0.05				
	Treatment Ⅱ	2.10 ± 0.05	2.33 ± 0.11	2.30 ± 0.05	2.45 ± 0.05	2.80 ± 0.07	2.64 ± 0.05	2.65 ± 0.11				
*p*-value		0.696	0.169	0.094	0.012	0.001	0.067	0.067				
T4 (nmol/L)	CON	39.53 ± 3.95	45.33 ± 1.14	37.18 ± 2.73	46.06 ± 3.06	45.50 ± 2.50	47.47 ± 2.03	48.87 ± 2.60	0.986	44.067, 0.000	22.530, 0.002	4.302, 0.052
	Treatment Ⅰ	35.04 ± 0.99	49.92 ± 1.98	43.68 ± 1.78	54.38 ± 2.98	65.57 ± 2.92	60.00 ± 3.69	61.21 ± 2.76				
	Treatment Ⅱ	43.75 ± 1.89	55.53 ± 2.67	57.68 ± 2.18	62.14 ± 2.34	70.55 ± 3.74	67.89 ± 3.48	68.31 ± 3.60				
*p*-value		0.112	0.019	0.001	0.009	0.000	0.004	0.004				
HSP70(pg/mL)	CON	120.23 ± 10.10	113.37 ± 6.16	114.08 ± 7.44	128.58 ± 9.50	126.99 ± 10.21	120.84 ± 6.32	120.84 ± 11.17	0.976	0.182, 0.836	3.440, 0.101	2.498, 0.148
	Treatment Ⅰ	104.70 ± 5.92	123.69 ± 10.86	122.65 ± 5.16	117.99 ± 3.82	113.81 ± 9.97	119.84 ± 5.17	128.03 ± 8.54				
	Treatment Ⅱ	115.08 ± 11.49	119.74 ± 9.21	121.43 ± 7.42	123.20 ± 6.60	121.77 ± 7.89	118.19 ± 5.56	113.04 ± 5.70				
*p*-value		0.523	0.722	0.815	0.733	0.526	0.508	0.508				

Note: Global analysis uses repeated measures analysis of variance. *p* < 0.05 has statistical significance (the same as below).

**Table 5:** Effects of astragalus polysaccharides on serum antioxidant indexes of dairy cows.

Item	Groups	Time(d)							Holistic Analysis (Huynh-Feldt)	Concentration (F, P)	Time (F, P)	Interaction (F, P)
		0	4	7	11	14	18	21				
SOD/(U·mL^−1^)	CON	48.39 ± 4.50	38.10 ± 4.01	41.91 ± 9.74	46.48 ± 3.30	57.78 ± 7.92	43.43 ± 10.40	43.43 ± 3.46	1.000	0.468, 0.641	0.692, 0.652	2.886, 0.115
	Treatment Ⅰ	42.29 ± 7.12	56.26 ± 8.97	50.79 ± 11.68	52.20 ± 3.91	45.72 ± 4.14	41.15 ± 7.94	42.14 ± 5.65				
	Treatment Ⅱ	45.84 ± 9.38	59.05 ± 11.29	50.80 ± 4.77	52.32 ± 4.77	33.53 ± 7.74	52.32 ± 12.67	50.11 ± 4.22				
*p*-value		0.738	0.231	0.602	0.709	0.092	0.737	0.737				
MDA/(nmol·mL^−1^)	CON	2.27 ± 0.34	1.80 ± 0.26	3.14 ± 0.37	3.90 ± 0.45	4.42 ± 0.55	3.17 ± 0.38	3.31 ± 0.90	0.468	0.572, 0.584	1.239, 0.410	1.789, 0.249
	Treatment Ⅰ	2.62 ± 0.50	3.02 ± 0.78	2.10 ± 0.24	3.90 ± 1.10	2.56 ± 0.46	3.85 ± 0.57	6.40 ± 1.07				
	Treatment Ⅱ	2.27 ± 0.11	2.91 ± 0.31	1.98 ± 0.58	3.60 ± 0.38	2.21 ± 0.23	3.13 ± 0.41	4.48 ± 0.75				
*p*-value		0.741	0.230	0.405	0.949	0.212	0.445	0.445				
GSH-Px/(U·mL^−1^)	CON	545.45 ± 63.03	604.36±48.11	516.00 ± 41.99	583.64 ± 73.78	520.37 ± 34.32	540.62 ± 38.94	507.27 ± 56.17	0.647	0.922, 0.432	9.421, 0.014	4.913, 0.039
	Treatment Ⅰ	562.91 ± 79.35	569.45 ± 58.56	690.55 ± 37.03	744.00 ± 66.38	502.91 ± 36.58	614.02 ± 27.04	614.18 ± 45.75				
	Treatment Ⅱ	580.36 ± 46.42	597.82 ± 60.39	524.73 ± 51.42	722.18 ± 33.07	427.64 ± 29.69	563.07 ± 25.91	544.36 ± 53.73				
*p*-value		0.930	0.897	0.065	0.182	0.793	0.768	0.768				
LDH/(U·L^−1^)	CON	433.75 ± 27.16	371.69 ± 16.03	428.92 ± 23.69	414.16 ± 17.04	447.59 ± 24.32	491.27 ± 13.66	451.55 ± 33.24	0.976	0.182, 0.836	3.440, 0.101	2.498, 0.148
	Treatment Ⅰ	334.14 ± 24.26	519.28 ± 26.24	462.95 ± 22.24	515.94 ± 19.17	460.64 ± 27.49	495.18 ± 15.35	465.18 ± 23.45				
	Treatment Ⅱ	429.22 ± 25.96	433.13 ± 22.48	385.84 ± 24.08	454.52 ± 9.77	446.98 ± 20.90	456.02 ± 10.44	451.66 ± 16.66				
*p*-value		0.349	0.298	0.561	0.940	0.957	0.542	0.542				

**Table 6:** OPLS-DA model parameter list.

Groups	N	R^2^X(cum)	R^2^Y(cum)	Q^2^(cum)	R^2^	Q^2^
Z *vs* Q	10	0.407	0.956	0.449	0.883	−0.447
H *vs* Z	10	0.312	0.985	0.609	0.934	−0.298
H *vs* Q	10	0.544	0.999	0.795	0.978	−0.110

Note: The number of principal components in modeling. N: number of samples. R^2^X (cum): cumulative interpretation rate of the model in the X-axis direction when modeling multivariate statistical analysis. cum: the cumulative result of several principal components. R^2^Y (cum): cumulative interpretation rate of the model in the Y-axis direction. Q^2^ (cum): cumulative prediction rate of the model. R^2^, Q^2^: The parameters of the response sort test are used to measure whether the model is over-fitting.

**Table 7:** Identification and screening of serum differential metabolites of dairy cows.

Metabolite name	Classification	Retention Time (min)	Quant Mass	Z *vs* Q	H *vs* Z	H vs Q
2-Picolinic Acid	Organic acid	6.28	136	ns	↓↓↓	↓
3-Aminoisobutyric Acid	Organic acid	9.38	102	ns	↓↓	↓↓↓
Alanine	Amino acid	7.48	116	ns	↓	↓
Catechol	Amine	11.4	254	↓	↑	ns
Gamma-Aminobutyric Acid	Organic acid	9.38	102	ns	↓↓	↓↓↓
Glucose	Carbohydrate	23.64	319	ns	ns	↓↓
Glucose-1-Phosphate	Carbohydrate	17.59	274	↑	ns	ns
Glutathione	Organic acid	8.63	282	↑	↑	↑↑↑
Glycerol-1-Phosphate	Organic acid	18.36	191	ns	ns	↑↑↑
Glycine	Amino acid	11.18	174	↑↑	ns	↑↑
Lysine	Amino acid	24.15	174	↑↑	ns	↑↑
Lyxitol	Carbohydrate	20.11	305	↓	ns	↓
Methylamine	Amine	15.68	176	↓	↑	ns
Nicotianamine	Alkaloid	7.54	187	ns	↓	↓
Norvaline	Amino acid	12.93	144	ns	↓	↓
Phenylacetic Acid	Organic acid	11.09	91	↓↓	↓↓↓	ns
Pyrophosphate	Inorganic acid	18.73	451	ns	↑	↑↑↑
Putrescine	Amine	13.76	174	↑↑	↑	↑↑↑
Tryptophan	Amino acid	28.8	202	↑↑	ns	↑↑↑
Tyrosine	Amino acid	24.46	218	↑	ns	↑

Note: ↑significant upregulation, ↓significant downregulation, ns: not significant, ↑: *p* < 0.05, ↑↑: *p* < 0.01, ↑↑↑: *p* < 0.001.

**Table 8:** The detailed results of potential metabolic pathways.

Potential Metabolic Pathway	−log(*p*)	Relevant Metabolites
Protein digestion and absorption	4.49	glucose, glycine, tryptophan, tyrosine
Glutathione metabolism	3.34	glycine, glutathione, putrescine
Pentose and glucuronate interconversions	2.87	glucose-1-phosphate, lyxitol
Glycolysis / Gluconeogenesis	2.15	glucose, glucose-1-phosphate
Biosynthesis of amino acids	1.83	glycine, tryptophan, tyrosine

Note: **−**log (*p*) is from the original *p*-value calculated from the enrichment analysis.

## Abbreviations

APS	Astragalus polysaccharides
COR	Cortisol
T3	Triiodothyronine
T4	Thyroxine
INS	Insulin
GC	Glucagon
HSP70	Heat shock protein 70
SOD	Superoxide dismutase
MDA	Malondialdehyde
GSH-Px	Glutathione peroxidase
LDH	Lactate dehydrogenase
GC-MS	Gas chromatography-mass spectrometry
TCA	Tricarboxylic acid
T	Temperature
RH	Relative humidity
THI	TEMPERATURE humidity index
Q	Pre-test
Z	Mid-test
H	Post-test
PCA	Principle component analysis
OPLS-DA	Orthogonal partial least-squares-discriminant analysis
VIP	Variable importance in the projection
FC	Fold change
GABA	γ-Aminobutyric acid
CDF	Common data format
KEGG	Kyoto encyclopedia of genes and genomes
MBROLE	Metabolites biological role
